# Religio Medici

**Published:** 1887-08-13

**Authors:** 


### Religio Medici.

Doctors are not, as a rule, given to formulating their creeds. The life of the true physician is in itself a religion, a ministry to the suffering of which the example was set by Christ Himself, and it is for most men so engrossing that in the active piety of their work they have no time to exercise their imaginations with the mysteries of dogma, much less to set down in writing their opinions on this or that detail of belief. This is the general experience of our day; and in the past only one doctor has been found who cared to analyse his creed and publish to the world the workings of his soul. That this book, the *Religio Medici* of Sir Thomas Browne, has survived to our day, and forms entertaining and instructive reading to others than antiquaries, is due not so much to the doctrine which the conscientious old "medicus" held or rejected as to the portrait of himself which we may read between the lines of his book.

Sir Thomas Browne was a doctor in Halifax. He finally removed his abode to Norwich, but the *Religio Medici* was written at Shipden Hall, near Halifax. The date of its composition is uncertain; in fact, we know but little of Browne's life, and have to guess at many circumstances from allusions in his works; but as he settled at Shipden in 1633, and several manuscript copies of the work were in existence before the publication of the first edition in 1642, it is surmised that the book was written between 1633 and 1638. He was the author of several other works—*Hydriotaphia*, *Quincunx*, *Pseudodoxia Epidemica*, etc.—all of which show a strange combination of shrewdness and credulity, of science and superstition; but the *Religio Medici* is the only one of his books which has much interest for a later generation. It became so popular immediately after its appearance that several imitations were published, and we have the *Religio Clerici*, *Religio Laici*, *Religio Militis*, and so forth, till one would think that every profession required a religion of its own. These works are for the most part uninteresting variations of Browne's work, and have fallen into peaceful oblivion, while his has attracted readers in every age.

Browne begins by the simple statement that he has a religion—a quite unnecessary piece of information, one would say, if the author did not go on to explain that there was a general tendency to believe that he had none. This impression he set down to three causes:—"The general scandal of my profession—the natural course of my studies—the indifferency of my behaviour and discourse in matters of religion (neither violently defending one, nor with that common ardour of contention opposing another)." It appears therefore that in the seventeenth century there was a widespread idea that doctors paid little attention to religion; that the study of natural science conduced to atheism; and that toleration, instead of being a Christian virtue, was only a mask for indifference—misconceptions which, unfortunately, are not exploded in the present day, and may yet exist for many a year. When all men *live* their religion, instead of merely talking about it, the need for formulated and circumscribed beliefs will cease, and at the same time will end those doubts of our neighbours' orthodoxy which usually tend, morally or physically, to arrangements *de hæretico comburendo*.

Tolerance is the most marked characteristic of our physician's character. He is proud to call himself a Christian. He says: "I find myself obliged, by the law of mine own reason, to embrace no other name than this"; but immediately adds, "neither doth herein my zeal so far make me forget the general charity I owe unto humanity, as rather to hate than pity Turks, Infidels, and (what is worse) Jews."

Carrying his definition further—"because the name of a Christian is become too general to express our faith, there being a geography of religion as well as lands"—he states that he belongs to that "reformed new-cast religion wherein he dislikes nothing but the name; of the same belief our Saviour taught, the Apostles disseminated, the fathers authorised, and the martyrs confirmed." But though a strict Protestant, and a member of the Church of England, he is by no means so out of sympathy with those Christians who belong to the Church of Rome, as were most of his fellow-countrymen in his time. "We have reformed from them, not against them," he says; "there is between us one common name and appellation, one faith and necessary body of principles common to us both;—and therefore I am not scrupulous to converse and live with them, to enter their churches in default of ours, and either pray with them or for them." He admits the errors into which their forms of worship may lead them, but values the devotional spirit that unites all souls. "I could never hear an Ave-Mary bell without an elevation," he tells us; "or think it a sufficient warrant, because they erred in one circumstance, for me to err in all,—that is, in silence and dumb contempt. Whilst, therefore, they directed their devotions to her, I offered mine to God; and rectified the errors of their prayers by rightly ordering my own."

He even rebukes the rash and intemperate language which was then commonly applied to the Pope,—"to whom as a temporal prince we owe the duty of good language," remarks this sturdy but courteous Protestant. "I confess there is a cause of passion between us," he admits; "by his sentence I stand excommunicated; heretic is the best language he affords me: yet can no ear witness I ever returned to him the name of antichrist or man of sin."

Frankly, the doctor does not like quarrelling. "I could never divide myself from any man upon the difference of an opinion, or be angry with his judgment, for not agreeing with me in that from which, perhaps, within a few days, I should dissent myself." There is discretion, and it may be laziness, as well as charity, in this tolerant spirit. "I have no genius for disputes," he remarks; and subsequently he says, shrewdly: "Every man is not a proper champion for truth, nor fit to take up the gauntlet in the cause of verity; many, from the ignorance of these maxims, and an inconsiderate zeal unto truth, have too rashly charged the troops of error, and remain as trophies unto the enemies of truth. A man may be in as just possession of truth as of a city, and yet be forced to surrender; 'tis therefore far better to enjoy her with peace, than to hazard her on a battle."

Yet Sir Thomas had his own struggles, though not with material or outside foes. He confesses to having cherished three heresies once on a time. The first of these, and the one he seems to consider most serious, would provoke but little interest in our minds,—namely, that the souls of men perished with their bodies, but should yet be raised again at the last day. Failing any direct knowledge of the subject, we might ask if it really mattered though the soul also should sleep in the grave for a time, if finally it might enjoy immortality; but though we cannot think there is much cause for Browne's anxiety on this matter, the reason he gives for his doubt is touching in its humility:—"A serious reflex upon my own unworthiness did make me backward from challenging this prerogative of my soul; so that I might enjoy my Saviour at the last, I could with patience be nothing almost unto eternity."

The second heresy he held was that of Origen, and, indeed, of gentle and tender souls in all ages: that God would not persist in His vengeance for ever, but, after a definite time of His wrath, would release the damned souls from torture. "Which error," says the doctor, "I fell into upon a serious contemplation of the great attribute of God, His mercy, and did a little cherish it in myself, because I found therein no malice, and a ready weight to sway me from the other extreme of despair, whereunto melancholy and contemplative natures are too easily disposed." Perhaps, though he does not own it, Browne felt tenderly towards this heresy to the full extent in which Origen held it, and which Burns, long afterwards, would gladly have shared, had the creed in which he was reared permitted it—that even the devil should one day be freed from torment.

The third error to which he pleads guilty—"I did never," he protests, "positively maintain as a practice, but have often wished it had been consonant to truth, and not offensive to my religion; and that is, the prayer for the dead; whereunto I was inclined from some charitable inducements, whereby I could scarce contain my prayers for a friend at the ringing of a bell, or behold his corpse without an orison for his soul. 'Twas a good way, methought, to be remembered by posterity, and far more noble than a history."

He does not, however, attempt to justify these inclinations of his, and declares that he never tried to propagate them; so pleads that they were hardly heresies, but mere errors, lapses of the understanding which did not involve any wilful depravity—an opinion which most of his modern readers will share. Indeed, they are likely to think the more kindly of the old physician, that "e'en his failings leaned to mercy's side."

(*To be continued.*)

